# Detection rates of clinically significant genomic alterations by microarray analysis for specific anomalies detected by ultrasound

**DOI:** 10.1002/pd.3943

**Published:** 2012-07-30

**Authors:** Lisa G Shaffer, Jill A Rosenfeld, Mindy P Dabell, Justine Coppinger, Anne M Bandholz, Jay W Ellison, J Britt Ravnan, Beth S Torchia, Blake C Ballif, Allan J Fisher

**Affiliations:** 1 Signature Genomic Laboratories, PerkinElmer, Inc.Spokane, Washington, USA; 2 Commonwealth Perinatal ServicesRichmond, Virginia, USA

**Keywords:** abnormal ultrasound, microarray, Array CGH < PRENATAL CYTOGENETICS, prenatal, congenital anomalies, soft markers

## Abstract

**Objective:**

The aim of this study is to understand the diagnostic utility of comparative genomic hybridization (CGH)-based microarrays for pregnancies with abnormal ultrasound findings.

**Methods:**

We performed a retrospective analysis of 2858 pregnancies with abnormal ultrasounds and normal karyotypes (when performed) tested in our laboratory using CGH microarrays targeted to known chromosomal syndromes with later versions providing backbone coverage of the entire genome. Abnormalities were stratified according to organ system involvement. Detection rates for clinically significant findings among these categories were calculated.

**Results:**

Clinically significant genomic alterations were identified in cases with a single ultrasound anomaly (*n* = 99/1773, 5.6%), anomalies in two or more organ systems (*n* = 77/808, 9.5%), isolated growth abnormalities (*n* = 2/76, 2.6%), and soft markers (*n* = 2/77, 2.6%). The following anomalies in isolation or with additional anomalies had particularly high detection rates: holoprosencephaly (*n* = 9/85, 10.6%), posterior fossa defects (*n* = 21/144, 14.6%), skeletal anomalies (*n* = 15/140, 10.7%), ventricular septal defect (*n* = 14/132, 10.6%), hypoplastic left heart (*n* = 11/68, 16.2%), and cleft lip/palate (*n* = 14/136, 10.3%).

**Conclusions:**

Microarray analysis identified clinically significant genomic alterations in 6.5% of cases with one or more abnormal ultrasound findings; the majority were below the resolution of karyotyping. Larger data sets such as this allow for sub-stratification by specific anomalies to determine risks for genomic alterations detectable by microarray analysis. © 2012 John Wiley & Sons, Ltd.

## INTRODUCTION

Invasive prenatal testing is used mainly to identify chromosome abnormalities in the fetus. The chance of finding a cytogenetic aberration depends on many factors, including family history of a chromosome abnormality, the age of the mother, and whether fetal anomalies are identified by ultrasound. In the case of fetal ultrasound anomalies, the specific structural finding or the involvement of more than one system appears to influence the chance of identifying an abnormal fetal karyotype.

Several studies have attempted to stratify the risk of a chromosome abnormality based on the specific fetal anomalies identified.^1^–^6^ Over all anomalies identified, the chance of finding an abnormal fetal karyotype ranged in these studies from ∼9% to ∼19%; the majority were aneuploidies and triploidies. After excluding these classes, the remaining chromosome abnormalities identified in one study (∼28% of the total abnormal karyotypes) were marker chromosomes, large deletions, and unbalanced translocations.^1^ In another study,^3^ 19.2% of fetuses (412/2143) had chromosomal abnormalities identified by conventional chromosome analysis, with the total number of non-aneuploid, abnormal fetal karyotypes at 1.2% of all cases studied and 6% of all abnormal karyotypes found. These findings prompted us to contemplate the number of submicroscopic genomic alterations present in fetuses with structural anomalies that are not identified by traditional karyotyping. The recent utilization of microarray analysis in prenatal testing allows for estimates of the frequency of identifying clinically significant copy number alterations (CNAs) by microarray analysis that are not detected by routine fetal karyotyping.

There have been many publications on the use of microarrays to identify clinically significant CNAs in a variety of prenatal samples,^7^–^16^ and several studies have focused specifically on the use of microarray analysis in prenatal diagnosis of fetuses with abnormal ultrasound findings.^17^–^23^ In these studies, about 8% to 16% of cases with normal routine karyotypes showed clinically significant genomic CNAs after microarray analysis, using various techniques and levels of reportable deletion and duplication size. Of further interest in prenatal diagnosis are the detection rates of submicroscopic genomic alterations for specific fetal anomalies. In most of these microarray studies, the number of cases investigated was too small to allow for meaningful stratification of the data to identify the risk of finding an abnormality by microarray with particular structural defects in the fetus. One exception is the publication by Lee and coauthors^16^ in which clinically significant CNAs were identified in 10.5% of fetuses with a single anomaly and in 15.4% of fetuses with two or more anomalies after a normal karyotype analysis. Further stratification of their data found detection rates after microarray analysis in fetuses with specific anomalies. However, their sample size of pregnancies with abnormal ultrasounds was still limited (*n* = 194).

In our studies, we have shown that 5.3% of pregnancies with any indication for study (IFS), and 6.5% of pregnancies with abnormal ultrasound findings, will have clinically significant CNAs identified by prenatal microarray analysis (Shaffer *et al*., accompanying article).^24^ In the current study, we have further stratified the data on 2858 cases with abnormal ultrasound findings to better understand the detection rates of clinically significant CNAs for specific anomalies identified by ultrasound examination.

## METHODS

Prenatal samples from amniotic fluid, chorionic villi, fetal blood, or products of conception were received by our laboratory from July 2004 through December 2011 for cytogenetic diagnosis using various microarrays targeted to known chromosomal syndromes with later versions providing backbone coverage of the entire genome (http://www.signaturegenomics.com/detection_rates.html). Although our laboratory participated in the Eunice Kennedy Shriver National Institute of Child Health & Human Development (NICHD)-sponsored clinical trial in prenatal microarray testing,^25^ none of the samples reported here were received as a part of that study. All data used in the analyses presented here were gathered or generated during the process of clinically approved microarray-based comparative genomic hybridization testing for routine patient care. Excluding samples that failed to generate results, a total of 5003 samples were tested for a variety of indications, including 2858 samples for abnormal ultrasound findings, which includes soft markers. Because of the increased likelihood of identifying CNAs, all cases with known abnormal fetal karyotypes at the time of microarray testing, with a family history of a chromosome rearrangement in a parent, and of fetal demise were excluded from the 2858 cases. Pregnancies therapeutically terminated because of ultrasound anomalies were not considered to be fetal demises and are included in this cohort.

Microarray analysis was performed as previously described.^9^ Results were reported to physicians as normal (no clinically significant CNA, with or without benign CNAs identified), unclear or uncertain, or clinically significant (abnormal). Each case was reviewed (by author JAR) and categorized according to the ultrasound abnormalities identified on the laboratory requisition form and ultrasound reports (when provided) and whether the test result was reported as normal, unclear, or clinically significant (abnormal). Cases with unclear results were further reviewed (by authors LGS and JAR) and in some cases, reassigned to the normal or abnormal groups as appropriate based on new knowledge gained from the medical literature and from our own experience since the initial reporting of the case. Ultrasound anomalies were categorized in several ways including multiple structural anomalies, structural anomalies limited to a single organ system, isolated abnormalities of growth, isolated abnormal amniotic fluid volume, single soft marker, multiple soft markers, or multiple nonstructural anomalies. Soft markers included choroid plexus cysts, echogenic foci in the heart or bowel, isolated short long bones, absent nasal bone, single umbilical artery, persistent right umbilical vein, sandal gap between the first and second toes, and fifth finger clinodactyly. Increased nuchal translucency (NT), increased nuchal fold, and cystic hygroma were categorized as structural anomalies in an abnormal body fluid category, which also included pericardial and pleural effusions, edema, ascites, and hydrops. For structural anomalies, cases were classified as involving single or multiple organ systems with or without additional nonstructural findings, such as intrauterine growth retardation, abnormal amniotic fluid volume, and/or soft markers (Table 1), and cases with and without additional nonstructural anomalies were combined for further detection rate calculations (Table 2). Detection rates for specific fetal anomalies were calculated if at least 20 cases were referred with the anomaly, either as an isolated finding or in association with other anomalies (Tables 2, 3). If it was not clear as to which category a case should be classified, it was further reviewed (by author AJF) and classified accordingly. Abnormal results were further stratified based on the size of the alteration (by author JAR). If the abnormality was an unbalanced translocation, the largest chromosomal segment affected by the translocation determined whether the case was placed in the >10 Mb category, for those CNAs detectable by karyotype, or the <10 Mb category, for those CNAs below conventional karyotype resolution (Table 2).

**Table 1:** Summary of microarray results in 2858 cases referred for abnormal ultrasound findings

	**Microarray results**			
		
**Ultrasound category**	**Normal (%)**	**Unclear (%)**	**Significant (%)**	**TOTAL**
**Structural abnormalities in multiple systems**	**492 (85.0)**	**29 (5.0)**	**58 (10.0)**	**579**
**Structural abnormalities in multiple systems + nonstructural anomalies: TOTAL**	**196 (85.6)**	**14 (6.1)**	**19 (8.3)**	**229**
+ IUGR	17 (77.3)	2 (9.1)	3 (13.6)	22
+ Abnormal amniotic fluid volume	34 (97.1)	0 (0.0)	1 (2.9)	35
+ Soft marker(s)	119 (83.8)	9 (6.3)	14 (9.9)	142
+ Multiple additional findings	26 (86.7)	3 (10.0)	1 (3.3)	30
**Structural abnormality(ies) in a single system: TOTAL**	**1370 (90.2)**	**68 (4.5)**	**81 (5.3)**	**1519**
CNS	286 (87.7)	17 (5.2)	23 (7.1)	326
Heart	182 (94.3)	5 (2.6)	6 (3.1)	193
Facial features	62 (88.6)	5 (7.1)	3 (4.3)	70
Respiratory	41 (85.4)	4 (8.3)	3 (6.3)	48
Gastrointestinal	8 (88.9)	0 (0.0)	1 (11.1)	9
Body wall	43 (82.7)	5 (9.6)	4 (7.7)	52
Genitourinary	61 (88.4)	5 (7.2)	3 (4.3)	69
Musculoskeletal	142 (86.1)	8 (4.8)	15 (9.1)	165
Neck and/or body fluids	544 (92.8)	19 (3.2)	23 (3.9)	586
Other: small thymus	1 (100.0)	0 (0.0)	0 (0.0)	1
**Structural abnormality(ies) in a single system + nonstructural anomalies: TOTAL**^a^	**220 (86.6)**	**16 (6.3)**	**18 (7.1)**	**254**
+ IUGR or overgrowth	47 (79.7)	4 (6.8)	8 (13.6)	59
+ Abnormal amniotic fluid volume	19 (86.4)	1 (4.5)	2 (9.1)	22
+ Soft marker(s)	120 (91.6)	7 (5.3)	4 (3.1)	131
+ Placental abnormality	3 (75.0)	0 (0.0)	1 (25.0)	4
+ Multiple additional findings	31 (81.6)	4 (10.5)	3 (7.9)	38
CNS	51 (91.1)	3 (5.4)	2 (3.6)	56
Heart	40 (90.9)	4 (9.1)	0 (0.0)	44
Facial features	15 (83.3)	0 (0.0)	3 (16.7)	18
Respiratory	1 (50.0)	0 (0.0)	1 (50.0)	2
Gastrointestinal	3 (60.0)	1 (20.0)	1 (20.0)	5
Body wall	3 (100.0)	0 (0.0)	0 (0.0)	3
Genitourinary	39 (84.8)	3 (6.5)	4 (8.7)	46
Musculoskeletal	31 (81.6)	4 (10.5)	3 (7.9)	38
Neck and/or body fluids	37 (88.1)	1 (2.4)	4 (9.5)	42
**Polyhydramnios or oligohydramnios, isolated**	**6 (66.7)**	**2 (22.2)**	**1 (11.1)**	**9**
**Abnormal growth, isolated**	**74 (97.4)**	**0 (0.0)**	**2 (2.6)**	**76**
**Single soft marker, isolated**	**55 (93.2)**	**2 (3.4)**	**2 (3.4)**	**59**
**Multiple soft markers, isolated**	**17 (94.4)**	**1 (5.6)**	**0 (0.0)**	**18**
**Multiple nonstructural anomalies**	**48 (98.0)**	**0 (0.0)**	**1 (2.0)**	**49**
**Other**^b^	**5 (100.0)**	**0 (0.0)**	**0 (0.0)**	**5**
**Not specified**	**51 (83.6)**	**6 (9.8)**	**4 (6.6)**	**61**
**TOTAL**	**2534 (88.7)**	**138 (4.8)**	**186 (6.5)**	**2858**

CNS, central nervous system; IUGR, intrauterine growth retardation.

a Cases in this category are broken down twice: once by the nature of the secondary finding and once by the organ system involved.

b Includes abnormal placentas, twin-to-twin transfusion, amniotic bands, and fetal stroke.

**Table 2:** Rates and sizes of significant CNAs among cases with fetal structural anomalies.

	Isolated structural anomaly^a^				In association with structural anomalies in other systems^b^				Totals			
			
	Significant findings				Significant findings				Significant findings			
									
System or malformation	<10 Mb	>10 Mb	Number tested	DR (%)	<10 Mb	>10 Mb	Number tested	DR (%)	<10 Mb	>10 Mb	Number tested	DR (%)
**Central nervous system**	19	6	382	6.5	19	16	317	11.0	38	22	699	8.6
Posterior fossa defects	4	1	74	6.8	5	11	70	22.9	9	12	144	14.6
Cerebellar hypoplasia	4	1	30	16.7	1	4	21	23.8	5	5	51	19.6
Dandy Walker malformation	1	0	44	2.3	4	7	43	25.6	5	7	87	13.8
Holoprosencephaly	5	3	53	15.1	0	1	32	3.1	5	4	85	10.6
Ventriculomegaly	3	0	84	3.6	7	3	88	11.4	10	3	172	7.6
Agenesis of the corpus callosum	0	2	45	4.4	2	0	24	8.3	2	2	69	5.8
Microcephaly	0	1	32	3.1	1	0	5	20.0	1	1	37	5.4
Hydrocephalus	3	0	66	4.5	1	1	34	5.9	4	1	100	5.0
Spinal defects	0	0	20	0.0	1	1	35	5.7	1	1	55	3.6
**Cardiovascular system**	5	1	237	2.5	26	14	343	11.7	31	15	580	7.9
Hypoplastic left heart	4	0	42	9.5	5	2	26	26.9	9	2	68	16.2
Tetralogy of Fallot	0	0	18	0.0	5	0	25	20.0	5	0	43	11.6
VSD	0	0	38	0.0	8	6	94	14.9	8	6	132	10.6
Dextrocardia/situs inversus	0	0	21	0.0	1	0	27	3.7	1	0	48	2.1
**Facial features**	5	1	88	6.8	14	6	188	10.6	19	7	276	9.4
Cleft lip and/or palate	5	0	55	9.1	8	1	81	11.1	13	1	136	10.3
Micrognathia	0	1	30	3.3	2	4	59	10.2	2	5	89	7.9
**Respiratory system**	3	1	50	8.0	2	4	63	9.5	5	5	113	8.8
Diaphragmatic hernia	3	1	48	8.3	1	4	50	10.0	4	5	98	9.2
**Gastrointestinal system**	2	0	14	14.3	1	1	66	3.0	3	1	80	5.0
TE fistula/absent stomach	1	0	3	33.3	0	0	22	0.0	1	0	25	4.0
**Body wall**	3	1	55	7.3	2	0	71	2.8	5	1	126	4.8
Omphalocele	3	1	49	8.2	1	0	57	1.8	4	1	106	4.7
**Genitourinary system**	6	1	115	6.1	13	6	238	8.0	19	7	353	7.4
Echogenic kidneys	1	0	4	25.0	1	1	20	10.0	2	1	24	12.5
Cystic kidney(s)	1	0	15	6.7	3	0	27	11.1	4	0	42	9.5
Kidney agenesis^c^	0	0	9	0.0	2	1	37	8.1	2	1	46	6.5
Ambiguous genitalia	1	0	21	4.8	1	1	33	6.1	2	1	54	5.6
Hydronephrosis or pyelectasis	1	0	24	4.2	2	2	71	5.6	3	2	95	5.3
**Musculoskeletal system**	18	0	203	8.9	17	10	327	8.3	35	10	530	8.5
Skeletal anomalies, excluding digital	8	0	60	13.3	5	2	80	8.8	13	2	140	10.7
Clubfeet or hands	8	0	59	13.6	8	3	135	8.1	16	3	194	9.8
Clenched hands	1	0	8	12.5	0	2	30	6.7	1	2	38	7.9
Digital anomalies: polydactyly, syndactyly, ectrodactyly	3	0	44	6.8	1	0	31	3.2	4	0	75	5.3
Arthrogryposis	0	0	28	0.0	2	1	30	10.0	2	1	58	5.2
Scoliosis	0	0	8	0.0	0	0	26	0.0	0	0	34	0.0
**Neck and/or body fluids**	20	7	628	4.3	12	11	219	10.5	32	18	847	5.9
Fetal hydrops, ascites, or edema; pericardial and/or pleural effusion	4	2	86	7.0	6	2	89	9.0	10	4	175	8.0
Cystic hygroma	6	4	232	4.3	6	6	70	17.1	12	10	302	7.3
Increased nuchal fold	4	0	39	10.3	0	0	35	0.0	4	0	74	5.4
Increased nuchal translucency	8	2	303	3.3	2	4	49	12.2	10	6	352	4.5
<4 mm	1	0	113	0.9	0	1	7	14.3	1	1	120	1.7
≥4 mm	6	0	96	6.3	0	2	12	16.7	6	2	108	7.4

CNAs, copy number alterations; DR, detection rate of clinically significant abnormalities by microarray; TE, tracheoesophageal; VSD, ventricular septal defect.

a Not all cases for each system are represented in its subcategories; cases may also be counted in multiple subcategories if the indication for study included multiple anomalies in the same system.

b Cases with multiple anomalies are counted in multiple categories, in whichever systems where anomalies are present.

c Includes both unilateral and bilateral kidney agenesis.

**Table 3:** Rates of significant CNAs among cases with nonstructural anomalies or soft ultrasound markers.

	Isolated anomaly			In association with structural anomalies			In association with other nonstructural anomalies			Totals		
				
Ultrasound finding	Significant findings	Number tested	DR (%)	Significant findings	Number tested	DR (%)	Significant findings	Number tested	DR (%)	Significant findings	Number tested	DR (%)
**Abnormalties of growth**	2	76	2.6	14	137	10.2	1	46	2.2	17	259	6.6
IUGR	2	74	2.7	14	136	10.3	1	41	2.4	17	251	6.8
**Oligohydramnios**	1	4	25.0	2	45	4.4	1	13	7.7	4	62	6.5
**Polyhydramnios**	0	5	0.0	3	45	6.7	0	7	0.0	3	57	5.3
**Soft marker(s)**	2	77	2.6	21	325	6.5	1	33	3.0	25	436	5.7
Single umbilical artery	1	18	5.6	14	132	10.6	0	13	0.0	15	163	9.2
Absent nasal bone	0	5	0.0	2	27	7.4	1	2	50.0	3	34	8.8
Short long bones	1	11	9.1	4	56	7.1	1	15	6.7	6	82	7.3
Choroid plexus cysts	0	7	0.0	2	35	5.7	0	11	0.0	2	53	3.8
Echogenic bowel	0	8	0.0	2	74	2.7	1	27	3.7	3	109	2.8
Intracardiac echogenic focus	0	6	0.0	1	40	2.5	0	12	0.0	1	58	1.7

CNAs, copy number alterations; DR, detection rate of clinically significant abnormalities by microarray; IUGR, intrauterine growth retardation

## RESULTS

A total of 2858 prenatal cases with documented fetal anomalies were received by our laboratory for prenatal testing using microarray analysis. The average (mean) maternal age at time of testing was 31.8 years; 44% of mothers were of advanced maternal age (≥35 years at delivery). The majority were tested on whole-genome, oligonucleotide-based arrays (*n* = 2161, 76%); the remaining were tested by bacterial artificial chromosome-based arrays, either with coverage of the whole genome (*n* = 506, 18%) or with targeted coverage (*n* = 191, 7%). Most cases tested had previously normal karyotypes (*n* = 2052, 72%); the remaining had karyotyping performed concurrently to microarray (*n* = 465, 16%) or had unknown or failed karyotypes (*n* = 341, 12%). Over all cases, 6.5% showed clinically significant array results, and 4.8% showed variants of unclear significance (Table 1). When only the cases with previously normal karyotypes are considered, the detection rate for significant CNAs is similar (128/2052, 6.2%), demonstrating that, in general, detection rates from the larger cohort represent the identification of clinically significant CNAs above that detected by karyotype analysis. A total of 61 cases were received by the laboratory with an IFS of unspecified ultrasound anomalies, and five were classified as having ‘other’ abnormalities not fitting into established categories; these 66 cases were excluded from further analysis but are tallied in Table 1. When considering the four main categories of abnormal ultrasound cases (single organ system, multiple organ systems, nonstructural, and other/not specified), the rate of unclear results were not dependent on IFS (*χ*^2^ = 5.82, *df* = 3, *p* = 0.12).

### Anomalies in a single organ system

Among those cases with a single organ system affected, 1519 cases showed a single anomaly or multiple anomalies in a single system, and 254 cases had structural anomalies in a single system plus additional findings such as abnormalities of growth, amniotic fluid volume, or soft markers. Of these cases, 5.3 and 7.1%, respectively, showed clinically significant CNAs after microarray analysis (Table 1); these rates were not significantly different from each other (*p* = 0.30, Fisher Exact test). Examining detection rates for anomalies limited to a single system where at least 20 cases were tested, the systems with the highest detection rates were musculoskeletal (8.9%), respiratory (8.0%), and body wall (7.3%) (Table 2). However, statistical analysis showed that the detection rates were not significantly different among the various systems (*χ*^2^ = 14.1, *df* = 8, *p* = 0.08). The highest detection rates for isolated, specific single anomalies were seen with cerebellar hypoplasia (16.7%), holoprosencephaly (15.1%), clubfeet or hands (13.6%), and skeletal anomalies (13.3%) (Table 2). Despite the high detection rate for some specific central nervous system (CNS) anomalies, the overall detection rate for isolated CNS findings was only 6.5%. Table 2 also identifies the size of the CNAs as >10 Mb or <10 Mb. For isolated structural anomaly cases with clinically significant CNAs, 82% (81/99) of the findings were smaller than 10 Mb in size and would likely not be identified by routine fetal chromosome analysis.

### Anomalies in multiple organ systems

Among those cases with anomalies in multiple organ systems, 9.5% (77/808) showed clinically significant CNAs (Table 1). Examining specific anomalies, when present with anomalies in other organ systems and where at least 20 cases were tested, the highest detection rates were seen with hypoplastic left heart (26.9%); posterior fossa defects (22.9%), including Dandy Walker malformation (25.6%) and cerebellar hypoplasia (23.8%); tetralogy of Fallot (20.0%); and cystic hygroma (17.1%) (Table 2). Table 2 also differentiates the size of the CNAs identified. For cases with multiple structural anomalies with clinically significant CNAs, 68% (52/77) had CNAs smaller than 10 Mb in size and would likely not be identified by routine fetal chromosome analysis. The detection rate of clinically significant CNAs was significantly higher for cases referred with anomalies in multiple organ systems when compared with those with anomalies limited to a single organ system (*p* < 0.001, Fisher Exact test).

### Soft markers

Table 3 shows the detection rates of clinically significant CNAs in the cases with soft markers and other nonstructural ultrasound findings. Collectively, cases with isolated soft marker(s) had a detection rate of 2.6%, whereas those cases with soft marker findings in association with other structural anomalies had a higher detection rate of 6.5%; this difference was not statistically significant (*p* = 0.28, Fisher Exact test). In addition, soft marker findings in association with other nonstructural anomalies such as abnormalities of growth or amniotic fluid volume had a detection rate of 3.0%. In total, 24 of 435 cases with a soft marker (5.5%) had a clinically significant CNA after microarray analysis. The detection rates for specific soft markers are shown in Table 3, with single umbilical artery (9.2%), absent nasal bone (8.8%), and short long bones (7.3%) having the highest detection rates. Although these soft markers are used to identify fetuses at risk for aneuploidies, 96% (23/24) of the significant CNAs identified by microarray were non-aneuploid, and 79% (19/24) were submicroscopic chromosome aberrations. Collectively, the detection rate among cases with isolated nonstructural anomalies (6/211, 2.8%) was significantly lower than the detection rate for multiple anomalies (77/808, 9.5%; *p* < 0.001, Fisher Exact test) but not statistically different from the rate for anomalies in single organ systems (99/1773, 5.6%; *p* = 0.10, Fisher Exact test).

## DISCUSSION

It is desirable to understand the genetic etiology of fetal anomalies; the largest contributing group is chromosome abnormalities, and among that group, aneuploidy, specifically trisomy 21, is the most common.^26^ However, as much as 6 to 28%^1^,^3^ of chromosome abnormalities identified in fetuses with anomalies detected by ultrasound are not one of the common aneuploidies or triploidies but rather represent other chromosomal anomalies such as marker chromosomes, unbalanced translocations, and large deletions visible through the light microscope. Thus, the goal of prenatal diagnosis is to identify any potential chromosome abnormality contributing to fetal pathology, not just trisomy 21. To accomplish this goal, higher resolution analysis of fetal chromosomes is needed, beyond the resolution of routine karyotyping of amniocytes and chorionic villi.

### Increased resolution provided by prenatal microarray testing

**Figure 1:**
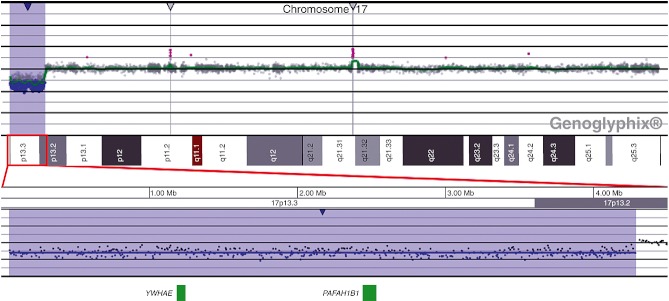
Identification of a 4.2-Mb deletion including the 17p13.3 Miller–Dieker syndrome region in a fetus with a 46,XX karyotype. Microarray plots show a single copy loss of 436 oligonucleotide probes from the terminal short arm of chromosome 17, identified in cultured amniocytes from a fetus referred with abnormal maternal serum screening (1/260 risk for open spina bifida) and subsequent fetal MRI that revealed decreased cerebral volume, short corpus callosum, and abnormal basal ganglia, giving the impression of migrational anomalies of the cortical and subcortical neural tissue. The deletion includes *YWHAE* and *PAFAH1B1 (LIS1)* (green boxes), associated with Miller–Dieker syndrome. Probes are ordered on the *x*-axis according to physical mapping positions, with the most distal 17p probes to the left and the most distal 17q probes to the right. Values along the *y*-axis represent *log*_2_ ratios of patient:control signal intensities. Results are visualized using Genoglyphix (Signature Genomics)

Although routine fetal chromosome analysis will identify a substantial proportion of fetuses with abnormal ultrasound findings with a cytogenetic aberration,^2^,^3^ given the limits in resolution of conventional chromosome analysis, most of these findings are trisomy 21 and other common aneuploidies and triploidies. The use of microarrays allows for the detection of both microscopic (aneuploidies and large structural rearrangements) and submicroscopic chromosome alterations. For cases with abnormal ultrasound findings, we have stratified the microarray data further based on a single anomaly, two or more anomalies, particular organ system involvement, and specific anomalies detected by ultrasound, along with the size of the clinically significant genomic alterations, to better understand the contribution of submicroscopic cytogenetic alterations to fetal pathology (Table 2). As our study mostly included fetuses with normal karyotypes, the majority of alterations identified were below the resolution of fetal karyotypes (<10 Mb). Among the cases with single structural anomalies, 82% (81/99) of significant CNAs were <10 Mb in size, and 68% (52/77) of significant CNAs in fetuses with multiple structural anomalies were <10 Mb; these abnormalities would be missed by conventional fetal karyotyping. Figure 1 shows an example of a case with microarray results below the resolution of routine karyotyping.

We have shown that additional, clinically significant CNAs are identified in 5.6% of fetuses with anomalies involving a single organ system and in 9.5% of fetuses with anomalies in multiple organ systems, which is comparable with the detection rates reported by Lee *et al*.,^16^ who found clinically significant CNAs in 10.5% of fetuses with a single anomaly and 15.4% in fetuses with two or more anomalies after a normal karyotype analysis. Staebler *et al*.^5^ provide the best attempt at defining detection rates for traditional karyotyping with a similar stratification of single and multiple anomalies. Assuming their study found all cytogenetically visible chromosome abnormalities, comparing our detection rates to their detection rates of 9% in cases with a single malformation and 19% in fetuses with multiple malformations show that microarray can offer a ∼50% increase in detection rate over traditional cytogenetic analysis in these pregnancies.

### Increased yield of CNAs in specific fetal anomalies

To understand the contribution of chromosome abnormalities to specific fetal anomalies, Staebler *et al*.^5^ reviewed the karyotypes of 428 fetuses examined because of fetal anomalies identified by ultrasound. Of these, 48 (11.2%) had abnormal karyotypes. Comparison of various anatomical systems among fetuses with single anomalies or single system involvement showed that the probability of an abnormal karyotype was significantly higher for a fetus presenting with hydrops or a cystic hygroma than a fetus with a CNS anomaly or a urinary tract anomaly.^5^ However, trisomies 21 and 18 accounted for 56% of the chromosome anomalies identified. There was no significant difference for the detection of chromosome anomalies between various organ systems for fetuses with multiple malformations. Among fetuses with isolated malformations, only four had chromosomal rearrangements (non-aneuploidy). Because of a bias towards aneuploidies and microscopically visible chromosome anomalies in their data set, caution must be exercised in interpreting the study's conclusions. The authors did not find any cytogenetic aberrations with certain single anomalies including hydronephrosis with high obstruction, unilateral multicystic dysplastic kidney, gastroschisis, intestinal dilatation or meconium peritonitis, cystic adenomatoid malformation, pulmonary sequestration, tumor, or vertebral anomaly.^5^ On the basis of these findings, the authors conclude that invasive procedures for these anomalies are questionable. However, our concern is that submicroscopic chromosome anomalies, missed by routine karyotyping, may account for some of these cases.

Among our cases, even though the numbers tested are small, we found the following detection rates among these ultrasound anomalies with ‘questionable’ support for invasive testing (Table 2): isolated hydronephrosis or pyelectasis (1/24, 4.2%, in a case with pyelectasis and megacystis), isolated vertebral anomalies (1/8, 12.5%, in a case with hemivertebrae), and isolated multicystic dysplastic kidney (1/15, 6.7%). Therefore, the finding of submicroscopic genomic alterations in these cases supports the use of arrays if invasive testing was desired. We did not detect any significant CNAs in fetuses with the following isolated anomalies, although our numbers are small: gastroschisis (0/3), intestinal dilatation (0/2), or cystic adenomatoid malformation (0/1). Furthermore, our data set did not have any fetuses found to have isolated pulmonary sequestration or tumors. Thus, larger data sets must be collected to further investigate the detection rates of submicroscopic chromosome abnormalities uncovered by microarray for these conditions.

In addition, although their number of cases was small (*n* = 194), Lee *et al*.^16^ were able to stratify their data into large anatomical system categories and found submicroscopic, significant abnormalities among fetuses with heart defects (7/50, 14%), cerebral anomalies (4/22, 18.2%), skeletal anomalies (2/23, 8.7%), gastrointestinal anomalies (1/7, 14.3%), and increased NT (1/17, 6.7%). These findings are similar to our data that also revealed substantial detection rates for congenital heart defects (46/580, 7.9%), CNS anomalies (60/699, 8.6%), musculoskeletal anomalies (45/530, 8.5%), gastrointestinal anomalies (4/80, 5.0%), and increased NT (16/352, 4.5%) with an isolated NT of ≥4 mm at 6.3% (6/96). Moreover, we identified significant CNAs in categories where their study had not, including isolated cleft lip/palate (5/55, 9.1%), isolated genitourinary malformations (7/115, 6.1%), isolated single umbilical artery (1/18, 5.6%), and isolated abnormal amniotic fluid volume (1/9, 11.1%). Although we have other categories where no significant CNAs have been identified, particularly with some isolated soft markers, relatively few cases have been referred for testing, preventing any definitive conclusions in this initial data set.

### A population perspective

Our detection rates of CNAs in the presence of certain ultrasound anomalies, in combination with the anomalies' population incidence, allow for predictions of the number of pregnancies that could receive specific genetic diagnoses if tested by microarray. For example, an incidence of hypoplastic left heart of approximately 1 in 5000 births^27^ and ∼4.13 million births annually in the USA^28^ means that approximately 825 affected babies are born each year in the USA; a total of ∼130 of these could receive a fetal diagnosis by microarray testing. A similar incidence is seen for posterior fossa defects,^29^ resulting in a potential diagnosis for another ∼120 pregnancies annually. Some malformations that are more common could lead to an even greater number of possible diagnoses, despite lower detection rates. For example, an incidence of ∼1 in 500 pregnancies for clubfeet^30^ could mean a potential diagnosis of another ∼800 pregnancies annually in the USA. These calculations are based on many assumptions, including that prenatal ascertainment is similar to birth rates and that the pregnancies tested in this study represent a unbiased sampling of pregnancies with these anomalies. Additionally, although it would not be expected that all such anomalies would be ascertained prenatally by ultrasound and all women would choose to have invasive diagnostic testing, this illustrates the unrealized potential for microarray testing to make thousands of diagnoses each year among fetuses with anomalies detected by ultrasound.

### Benefits and uncertainty in microarray testing

Because cytogenetic anomalies may be cryptic or not visible through the light microscope, but identifiable by microarray, the identification of a chromosome abnormality in the fetus allows for an informed investigation of parental karyotypes, increased understanding of the etiology of the fetal anomalies, and additional information for better genetic counseling of the couple for future risk management. Information obtained through microarray testing may be useful by allowing a more complete understanding of the prognosis for the fetus and may assist in decisions about pregnancy management. Thus, although some anomalies, such as holoprosencephaly, have a poor prognosis or may sometimes be considered lethal, establishing a genetic etiology is important for the family. Obtaining this information must be weighed against the chance of identifying variants of unclear clinical significance (Table 1 and Shaffer *et al*., accompanying article).^24^

### Limitations of our study

One caveat of this type of analysis is that it is dependent on information provided on laboratory requisition forms at the time of testing, and the large number of referral sites prevents consistency among the definition of particular ultrasound anomalies. Furthermore, postnatal confirmation of ultrasound anomalies was not available to us. In some cases, additional ultrasound anomalies may be present that were not included in the IFS. This would lead to an overestimation of detection rates for isolated anomalies and is a possible explanation why, in some categories, the detection rates for isolated anomalies are higher than those with multiple anomalies (for example, isolated clubfeet had a 13.6% detection rate, whereas in association with anomalies in other systems the rate was only 8.1%). On the whole, however, we showed that the detection rate for multiple anomalies was significantly higher than the rate for isolated anomalies, which indicates that much of the information provided in the IFS is likely accurate. There are additional limitations to certain IFS, such as ventriculomegaly and pyelectasis, which are considered soft markers when mild. In this study, IFS of ventriculomegaly and pyelectasis were typically not specific enough to distinguish between mild or severe, so they were all counted as structural abnormalities. Conversely, we were able to stratify increased NT on the basis of specific measurements for a subset of these cases (Table 2).

## CONCLUSION

This study has attempted to understand the specific detection rates for chromosome aberrations after microarray analysis for certain fetal anomalies. In some cases, the number of referrals or the number of abnormalities identified were too small to derive recommendations for specific anomalies. However, the goal of invasive prenatal testing is to identify fetal chromosome anomalies when present. We have demonstrated a significantly increased detection of chromosome anomalies after applying microarray analysis in all indications for prenatal testing (Shaffer *et al*., accompanying article).^24^ This is especially true in situations when an ultrasound examination reveals fetal anomalies (up to an overall 6.5% gain over chromosome analysis in this study), which supports the opinion of the American College of Obstetrics and Gynecology (ACOG) to endorse the use of microarray testing in the presence of fetal anatomic anomalies.^31^ Nicolaides and Snijders^1^ and ACOG^32^ advise that even when the risk of identifying a chromosome abnormality is low, women should be offered diagnostic testing to examine the fetal karyotype. The finding of a chromosome aberration provides more information for pregnancy and neonatal clinical management, and the rates reported in this study can provide better estimates of the likelihood of a submicroscopic chromosome aberration with specific ultrasound anomalies. Thus, for most informed medical management, pregnancies with ultrasound anomalies undergoing invasive testing should be tested by microarray to identify all clinically significant CNAs.

**WHAT'S ALREADY KNOWN ABOUT THIS TOPIC?**

- Microarray testing has the ability to detect large and small, clinically significant copy number alterations in pregnancies with abnormal ultrasound findings.

**WHAT DOES THIS STUDY ADD?**

- Through the analysis of a large retrospective data set, detection rates of microarray testing for various, specific abnormal ultrasound findings have been determined.
